# Characterization of *Pseudomonas stutzeri* strain AY4 with nitrogen removal and application in wastewater treatment

**DOI:** 10.1128/spectrum.03356-24

**Published:** 2025-08-26

**Authors:** Weilai Fu, Xiaolong Li, Peifeng Duan, Youxi Zhao

**Affiliations:** 1 Biochemical Engineering College, Beijing Union University 70541, Beijing, China; 2 Jiangsu Yuepeng Technology Co. Ltd, Yancheng, China; 3 Jiangsu Aishengmu Feed Co. Ltd, Yancheng, China; Institute of Microbiology, Chinese Academy of Sciences, Beijing, China

**Keywords:** *Pseudomonas stutzeri*, ammonium and nitrate nitrogen removal, farm wastewater treatment, transcription level differences

## Abstract

A new strain capable of removing ammonium and nitrate nitrogen from animal farm wastewater was isolated and identified as *Pseudomonas stutzeri* strain AY4. Nitrogen balance analysis showed 52.9% and 63.2% of NH_4_^+^-N and NO_3_^−^-N conversion to gaseous nitrogen, respectively, in laboratory conditions. Strain AY4 was also able to remove 92.7%, 95.8%, and 91.4% of total nitrogen, NH_4_^+^-N, and NO_3_^−^-N in the real farm wastewater, which was significantly greater than that of the uninoculated control group. The quantitative real-time PCR experiment showed that the transcript levels of *glnA*, *glnE*, *amtB*, *napA*, *narI*, *nirB*, and *nirD* genes related to nitrogen metabolism were significantly upregulated during wastewater treatment. Strain AY4 showed high potential working in low carbon-to-nitrogen ratio and wide range of temperature adaptability, which makes it suitable for wastewater treatment in various environmental conditions. In addition, the study of transcript levels of genes related to nitrogen metabolism is important for revealing the mechanism of action of *P. stutzeri* in wastewater treatment.

**IMPORTANCE:**

The heterotrophic nitrifying-aerobic denitrifying bacterium *Pseudomonas stutzeri* strain AY4, isolated in this study, simultaneously removed ammonium nitrogen and nitrate nitrogen from wastewater. Upregulation of the transcript levels of *glnA*, *glnE*, *amtB*, *napA*, *narI*, *nirB*, and *nirD* genes associated with nitrogen metabolism favored the removal of nitrogen from actual wastewater. The isolation of novel strains with nitrogen removal ability has positively contributed to the treatment of nitrogen-containing farm wastewater.

## INTRODUCTION

Large amounts of nitrogen accumulation due to the industrial and agricultural revolution have become one of the key factors disturbing ecological balances and environmental security. In agricultural practices, a significant quantity of nitrogen-containing fertilizers is being used, where a predominant portion of it remains unabsorbed by the plants. These redundantly surplus fertilizers then find their way into the rivers, lakes, rainwater, and stored groundwater, causing severe water pollution (1). Large amounts of untreated manures generated by animal breeding farms also contribute toward excessive nitrogen accumulation in the ecosystem (2). Excessive nitrogen concentration in water bodies causes eutrophication — the excessive quantity of algal bloom ultimately affecting the health of aquatic animal and marine ecosystems (3). This threat not only includes the safety of river and lake ecosystems but also jeopardizes the safety of shallow and deep groundwater systems (4).

The process of nitrification and denitrification is a crucial part of the nitrogen cycle in nature and is essential for maintaining mineral and material balance toward a healthy ecological development (5). Ammonium nitrogen is converted to nitrite by nitrifying bacteria under aerobic conditions and then converted to nitrate, explicating the oxidation process of nitrogen (6). Autotrophic nitrification includes both ammonia-oxidizing and nitrite-oxidizing bacteria. Denitrifying bacteria convert nitrates into gaseous nitrogen under anaerobic conditions, thereby achieving a reduction of nitrogen sources in the aquatic environments (7). Traditional nitrifying and denitrifying bacteria flourish very slowly and require an extended period of time for conversion and removal of nitrogen, thereby limiting their applications in nitrogenous wastewater treatment. Heterotrophic nitrifying-aerobic denitrifying microorganisms (HNADMs) are capable of removing ammonium and nitrate nitrogen simultaneously from the environment under aerobic conditions using energy provided by an organic carbon source (6). Organic carbon replaces inorganic carbon to satisfy the needs of bacterial growth. Oxygen not only provides energy for bacterial growth but also serves as an acceptor of electrons in the nitrogen denitrification process to replace nitrate nitrogen, showing greater dominance in wastewater treatment (8). To date, a wide range of HNADMs has been identified, including *Acinetobacter* (9, 10), *Sporidiobolus pararoseus* (11), *Bacillus* (12, 13), *Alcaligenes* (14, 15), *Klebsiella* (16, 17), *Paracoccus* (18), *Pseudomonas* (19, 20), and *Serratia* (21, 22).

The traditional nitrogenous wastewater treatment needs aerobic and anaerobic reaction tanks to facilitate effective nitrogen removal. Unlike the traditional treatment methods, nitrogen removal by HNADMs is carried out under aerobic conditions. One of the isolated *Pseudomonas stutzeri* SDU10 showed superior nitrogenous wastewater management ability with ammonium and chemical oxygen demand (COD) removal of 97.6% and 94.2%, respectively, from swine farm wastewater (23). The application of quantitative real-time PCR (qRT-PCR) study further reveals the changes in gene transcription levels, enlightening denitrification mechanism in HNADMs (24). These changes in transcription level can affect enzyme synthesis, which eventually rheostats the nitrogen removal efficiency. Thus, studying those changes might have a better role in the optimization of the wastewater treatment process by HNADMs.

As traditional nitrogenous wastewater treatment methods are expensive, time consuming, and less effective, microbial treatment methods have the advantage of being sustainable from both economic and time management perspectives. In this standpoint, HNADMs might appear as a redeemer in terms of eco-friendly farm wastewater treatment. This manuscript deals with an HNADM strain identified as *Pseudomonas stutzeri* strain AY4, capable of removing both ammonium and nitrate nitrogen simultaneously. The culture conditions were optimized to improve the nitrogen removal ability by AY4. Further application in real farm wastewater treatment and nitrogen metabolic transformation mechanism showed promising results. These findings shall certainly resolve the issue of traditional nitrogen effluent treatment cost as well as fix the environmental pollution issue due to eutrophication. In short, from the aspect of novelty, a successful attempt has been made to explore the potential of an HNADM strain in real farm wastewater treatment, with bacterial qRT-PCR data predicting its metabolic transformation trails for bridging the gap regarding nitrate-nitrite conversion understanding during nitrogen removal from farm wastewater environments by microbial community.

## MATERIALS AND METHODS

### Sample acquisition

The samples of the isolated strains were obtained from the wastewater treatment pond of a pig farm in Cangzhou, China.

### Medium component

Nitrification medium (NM) and denitrification medium (DM) were prepared according to previous literature, and minor modifications were made according to the actual nitrogen concentration requirements of the experiments (25). NM has the following composition (per liter): NH_4_Cl (0.76 g), C_4_H_4_Na_2_O_4_ (4.05 g), MgSO_4_·7H_2_O (0.1 g), NaCl (2.0 g), and KH_2_PO_4_ (0.5 g); initial pH was adjusted to 7. DM has the following composition (per liter): NaNO_3_ (1.22 g), C_4_H_4_Na_2_O_4_ (4.05 g), MgSO_4_·7H_2_O (0.1 g), NaCl (2.0 g), and KH_2_PO_4_ (0.5 g), pH was adjusted to 7. NM and DM were additionally supplemented with 2 mL of trace element solution, which was prepared with reference to the literature (25). The solid NMs and DMs were prepared by adding 2% agarose to it. Liquid and solid medium were both used after sterilization at 121°C for 30 minutes.

### Strain enrichment and isolation

One milliliter of well homogenized wastewater sample was added to 100 mL of NM and incubated for 4 days at 30°C with 180 rpm agitation. One milliliter of NM enrichment culture was aspirated and mixed into 100 mL of DM, followed by incubation for 4 days under the same conditions. The culture was incubated alternately in NM and DM five times to acclimatize the cells, which would then be able to utilize both nitrite and ammonium nitrogen. Finally, 0.1 mL of enrichment medium was spread evenly on solid DM plates and incubated at 30°C for 24 hours, with the plates kept inverted. Fast-growing single colonies were selected from the plates to check their ability to remove ammonium and nitrate. The 16S rRNA gene of the desired strains was amplified using the bacterial primer (27F/1492R), and the phylogenetic tree was constructed by using MEGA version 7.0 software (neighbor-joining tree method) by sequence comparison with the NCBI database and BLAST.

### Analytical methods

Concentrations of total nitrogen (TN), NH_4_^+^-N, NO_2_^−^-N, NO_3_^−^-N, and NH_2_OH-N in the medium were calculated by measuring the absorbance at specific wavelengths after the individual color development reaction (21, 26). The amount of intracellular nitrogen (intracellular-N) synthesized during the bacterial growth was estimated by dry cell mass weight after using C_5_H_7_NO_2_. Free organic nitrogen (organic-N) in the medium was calculated according to the following equation:

Organic-N = TN-(NH_4_^+^-N+NO_2_^−^-N+NO_3_^−^-N+NH_2_OH-N).

Gaseous nitrogen (gaseous-N), which includes NO, N_2_O, and N_2_ produced by HNADMs during denitrification, was calculated as follows:

Gaseous-N = *C*_0_ (initial nitrogen addition) − Ct (TN) − Ct (intracellular-N).

The equation of nitrogen removal rate was

N removal rate = (*C*_0_ − *C*_*t*_) / *C*_0_ × 100%.

*C*_0_ was the initial nitrogen concentration, and *C*_*t*_ was the final nitrogen concentration.

Bacterial growth was measured spectrophotometrically by detecting the absorbance of the culture medium at 600 nm.

### Nitrogen removal characteristics

NM (NH_4_^+^-N, 200 mg/L) was inoculated with the AY4 strain, and samples were collected every 12 hours to analyze the levels of TN, NH_4_^+^-N, NO_3_^−^-N, NO_2_^−^-N, and NH_2_OH-N and bacterial cell density (optical density at 600 nm [OD_600_]), and to plot the nitrification curve. The denitrification curve for strain AY4 in DM (NO_3_^−^-N, 200 mg/L) was plotted using the same sampling and analytical methods.

### Nitrogen balance analysis

Strain AY4 was inoculated in NM and DM to analyze the nitrogen transformation and removal capacity, respectively. Supernatant was collected by centrifugation (8,000 rpm, 10 minutes) after 96 hours of incubation, and TN, NH_4_^+^-N, NO_3_^−^-N, NO_2_^−^-N, NH_2_OH-N, and organic-N concentrations were measured. Cells collected after centrifugation were repeatedly washed three times using deionized water and then dried to reach a constant weight, and thus, intracellular-N was estimated (27).

### Nitrogen removal optimization

Two hundred fifty milliliter conical flasks were filled with 100 mL of NM or DM followed by incubation with the AY4 strain. Nitrogen removal and bacterial cell growth (OD_600_) were measured after 96 hours of cultivation. Carbon source type, carbon-to-nitrogen ratio (C/N), initial pH, incubation temperature, and agitation rate were optimized to enhance the denitrification ability by strain AY4. The five different carbon sources selected for the experiment were C_6_H_12_O_6_ (glucose), C_4_H_4_Na_2_O_4_ (sodium succinate), C_6_H_5_Na_3_O_7_ (sodium citrate), C_2_H_3_NaO_2_ (sodium acetate), and C_3_H_3_NaO_3_ (sodium pyruvate). C/N was set at five gradients, i.e., 4, 6, 8, 10, and 12. Gradients of initial pH were 5, 6, 7, 8, and 9. The incubation temperatures (°C) were set at four gradients as follows: 10, 20, 30, and 35. To vary the dissolved oxygen (DO) concentration of the medium, assuming it is intimately intertwined with the shaker speed, four different agitation speeds (rpm) were selected for the experiment, i.e., 120, 150, 180, and 210.

### Real wastewater treatment

The efficiency of the AY4 strain for removing nitrogen and COD from farm wastewater was measured in the laboratory. A 100 mL untreated farm wastewater sample was taken in a 500 mL conical flask and then inoculated with 5% (vol/vol, OD_600_ = 2.0) AY4 strain, followed by 96 hours incubation. In order to favor the growth of AY4, glucose was added to make C/N = 6.0 and pH = 7.0 for the wastewater; same alterations were made for the control and experimental groups. Samples were taken every 24 hour interval for TN concentration, OD_600_, and COD measurements.

### PCR and qRT-PCR verification

In order to find the possible underlying mechanism behind the wastewater treatment by AY4, changes in transcript levels of genes for enzymes associated with nitrogen metabolism were detected using qRT-PCR. Primers (Tables 1 and 2) were designed, and qRT-PCR was performed. Obtained data were analyzed and determined using the 2^−ΔΔ Ct^ model (24).

**TABLE 1:** PCR primers used in this work

Primer	Sequence (5′−3′)
*glnA*-F	ATGTCGAAGTCGCTTCAACT
*glnA*-R	TCAGACGCTGTAGTACAGAT
*glnE*-F	ATGAGTCTGCCGTCGCT
*glnE*-R	TCAGCTGAGCCCCA
*amtB*-F	ATGGAAAACCTCAACAGCG
*amtB*-R	TCAGTCGTGGCTGAG
*napA*-F	ATGAGCCTTACCCGACG
*napA*-R	TCAGGCGATGCTGACCA
*narI*-F	ATGTCTAACTTCAATTTCCTGC
*narI*-R	TCATACCGGGCTCTTGC
*narH*-F	ATGAAGATTCGTTCACAAGTAGG
*narH*-R	TCAGTCCTCCCACAGCT
*nirB*-F	ATGAGTTCAACCGTCACC
*nirB*-R	TCAGAGCACCTCCTGG
*nirD*-F	ATGAACTGGCTCGATATCTG
*nirD*-R	TCAGGCCGCGCATT
*norZ*-F	ATGAGCGACAAGAGCAAGAA
*Nor*Z-R	TCAGGCCGGTTCGA
*norR*-F	ATGATGGCTGATGCCCT
*norR*-R	CTACTTGAGCTTCAGCCG

**TABLE 2:** qRT-PCR primers used in this work

Primer	Sequence (5′−3′)
*glnA*-F	GTGAAGTGGATTGATCTGCG
*glnA*-R	CATCAGGATCATGTCGGAGG
*glnE*-F	AGGTCGAGTTTATCGCTCAG
*glnE*-R	GCAGGAATTCATAGCCTTCG
*amtB*-F	CATGGCTCCAATACCCTGTT
*amtB*-R	TAGCCGATGAAGAAGTACGC
*napA*-F	TCCTCAAACCCTACACCCTA
*napA*-R	TAGATCATGTTGTTCGCCCA
*narI*-F	TTCGACCTGTCGCAATACAG
*narI*-R	GATGAAGTGGTGATACAGCG
*narH*-F	GTTCGACTTCGACTACCAGA
*narH*-R	GTCGAAGTTCTTGTCCTTGC
*nirB*-F	ACATGTCGACCAAGCTCAA
*nirB*-R	TAGTAGCTGTTGTCACCGAC
*nirD*-F	TATCTGCGCACTGGATGAGA
*nirD*-R	GATCGATCTGCCAGTTGTGC
*norZ*-F	CGACCAGATCAAGACCAAGA
*Nor*Z-R	ACTCCTGAACACCATAGCTC
*norR*-F	ATCATCGCGGCGACTAAC
*norR*-R	GAGTTCGAGAAAATGCCCG

### Data analysis

The experimental test data were obtained in triplicate and analyzed using SPSS (v.23.0.0.0). Significance between experimental groups was analyzed by one-way analysis of variance and Duncan’s multiple comparison test and marked with superscript letters (*P* < 0.05).

## RESULTS

### Strain classification and identification

**Fig 1:**
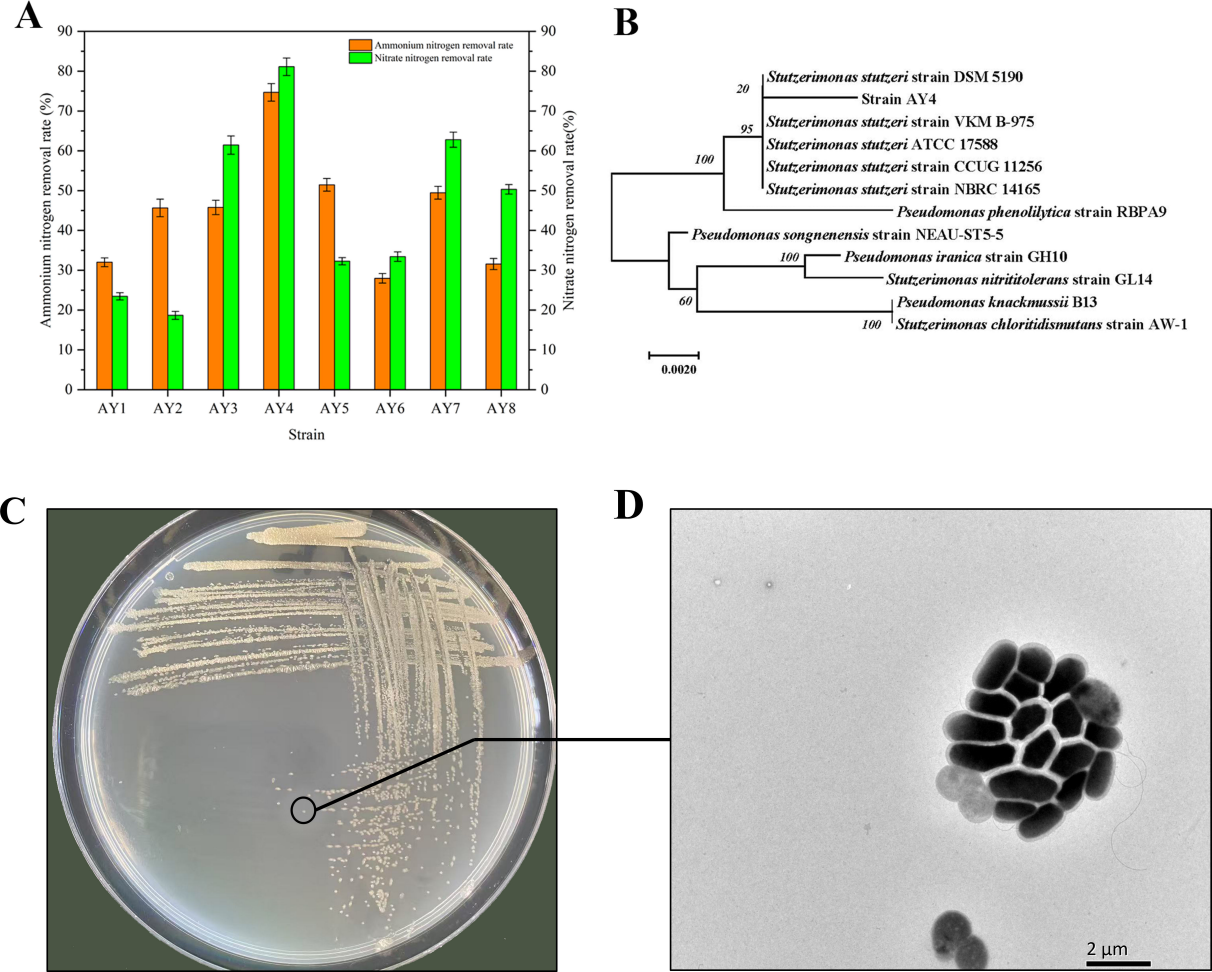
Screening of strain AY4 and phylogenetic tree based on 16S rRNA sequences. (**A**) Strain isolation and screening, (**B**) phylogenetic tree of strain AY4, (**C**) colony image of strain AY4, and (**D**) electron micrograph (SEM) of strain AY4.

Eight strains capable of ammonium and nitrate transformation were isolated from the collected samples, and strain AY4 was found to possess the highest nitrogen-transforming efficiency (81.1%) (Fig. 1A). The colony morphology of AY4 appeared to have an opaque, round, raised center and moist surface, with irregular margins. BLAST sequence comparison of AY4 16S rRNA in the NCBI database (accession number PQ319790) showed 99.4% similarity with *Pseudomonas stutzeri* strain ATCC 17588 (Fig. 1B).

### Nitrogen balance analysis

NM was supplemented with 208.2 mg/L of NH_4_^+^-N and inoculated with AY4 for 96 hours, and then inorganic nitrogen, organic nitrogen, and intracellular-N content were evaluated. As shown in Table 3, 159.3 mg/L of initial NH_4_^+^-N was converted to other forms of nitrogen after 96 hours of incubation, and the removal of NH_4_^+^-N reached 76.5%. Meanwhile, 110.3 mg/L of gaseous-N was generated during the cultivation process, which indicates 52.9% initial NH_4_^+^-N conversion toward gaseous-N. After 96 hours of incubation in DM inoculated with strain AY4 with NO_3_^−^-N as the nitrogen source, the removal of NO_3_^−^-N and the generation of gaseous-N reached 82.8% and 63.2%, respectively. Strain AY4 showed the ability to utilize NH_4_^+^-N and NO_3_^−^-N simultaneously under aerobic conditions similar to previously reported *Alcaligenes* sp. (28).

**TABLE 3:** Nitrogen balance analysis of strain AY4^*a*^

Initial nitrogen concentration (mg/L)		Final nitrogen concentration (mg/L)						Nitrogen removal rates (%)
		NO_2_^−^-N	NO_3_^−^-N	NH_4_^+^-N	Organic-N	Intracellular-N	Gaseous-N	
NH_4_^+^-N	208.22 ± 3.66	0.00 ± 0.01	25.83 ± 0.64	48.92 ± 2.32	4.36 ± 0.43	18.76 ± 1.02	110.35 ± 3.55	76.51 ± 1.68
NO_3_^−^-N	199.73 ± 4.05	13.8 ± 2.01	34.19 ± 1.23	1.05 ± 0.08	6.78 ± 1.16	17.66 ± 1.55	126.25 ± 3.98	82.88 ± 1.68

^*a*^ All experimental test data were replicated three times and data were marked by mean ± standard error (SE). Organic-N, organic nitrogen concentration; Intracellular-N, intracellular nitrogen concentrations; Gaseous-N, gaseous nitrogen concentration.

### Ammonium nitrogen removal

**Fig 2:**
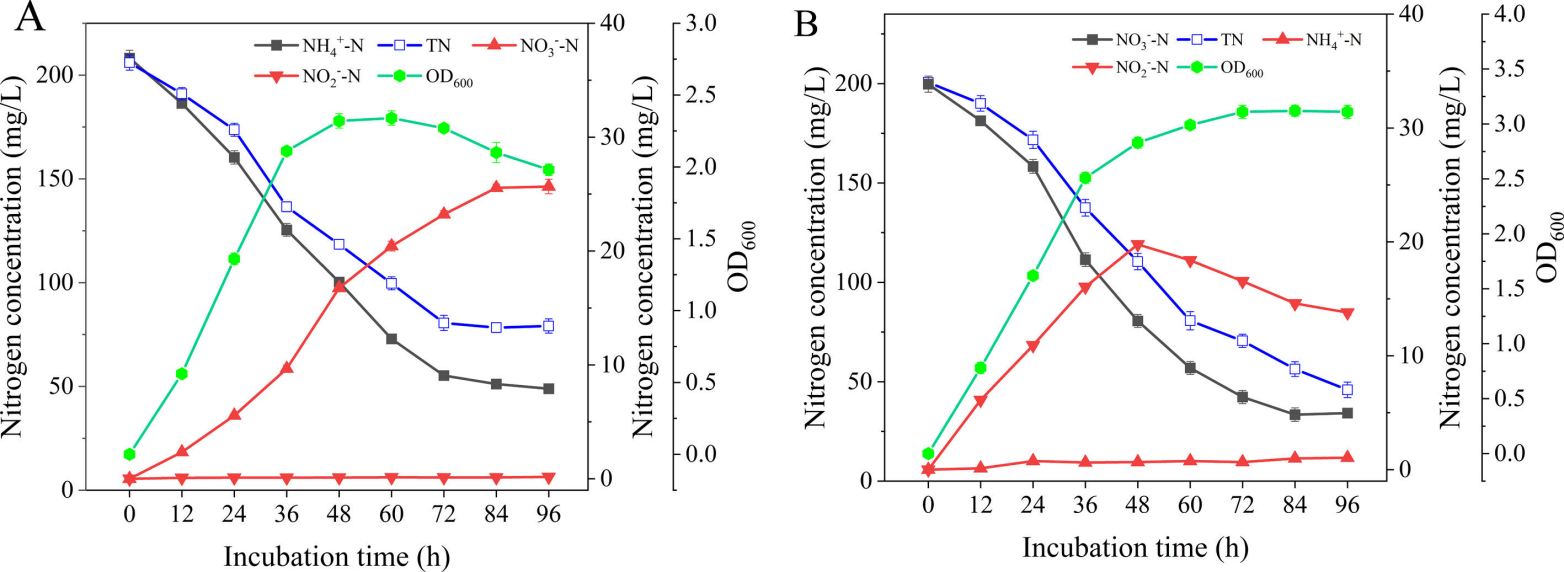
Characterization of nitrogen transformation and growth curve of strain AY4. (A) NH_4_^+^-N as nitrogen source (200 mg/L) and (B) NO_3_^−^-N as nitrogen source (200 mg/L). Data were analyzed using Duncan’s multiple comparison test. Different superscript characters in the bars indicate significant differences (*P* < 0.05).

When NH_4_^+^-N was supplemented to the NM as the sole nitrogen source, it was found that the cell density of AY4 increased rapidly during 12–36 hours and the growth stabilized after 48 hours, but then the OD_600_ value began to decrease until 72 hours (Fig. 2A). The concentration of NH_4_^+^-N decreased rapidly from 186.3 mg/L (12 hours) to 72.9 mg/L (60 hours), with a reduction rate of 2.4 mg/L/h. At the end of the incubation, NO_3_^−^-N levels started to rise steadily up to 25.6 mg/L and low levels of retention were observed afterward, while no hydroxylamine production was detected throughout the incubation.

### Nitrate nitrogen removal

The growth pattern during nitrogen transformation after inoculation with strain AY4 was also investigated when NO_3_^−^-N was used as the sole nitrogen source (Fig. 2B). At identical concentrations of added nitrogen source, NO_3_^−^-N was more favorable than NH_4_^+^-N for the growth of AY4, and the bacterial concentration (OD_600_) reached 3.1, which was much higher than that of 1.9 when NH_4_^+^-N was used as the nitrogen source. During the growth of AY4, NO_3_^−^-N concentration decreased continuously, but NH_4_^+^-N concentration increased steadily for 48 hours and then started to decrease. With regard to the nitrification process, NO_2_^−^-N concentration during denitrification was consistently low.

### Nitrogen removal optimization

#### Carbon source

**Fig 3:**
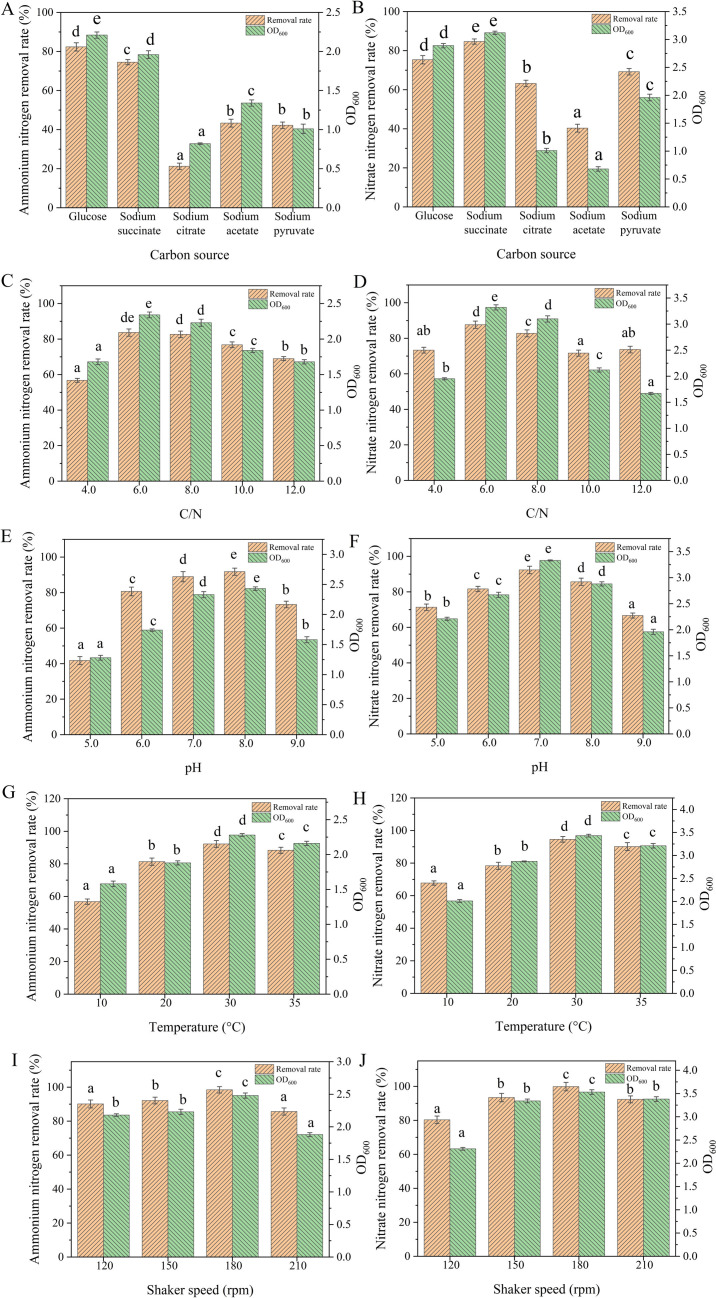
Optimization of conditions for ammonium and nitrate nitrogen removal by strain AY4. (**A **and **B**) Carbon source, (**C **and **D**) C/N, (**E **and** F**) initial pH, (**G** and **H**) temperature, and (**I **and **J**) shaker speed. Data were analyzed using Duncan’s multiple comparison test. Different superscript characters in the bars indicate significant differences (*P* < 0.05).

A suitable carbon source not only fulfills the growth requirements of any heterotrophic nitrifying bacteria but also contributes to nitrogen conversion efficiency amendment (7). Hence, the selection of the optimal carbon source is critical for strain AY4 to establish it as a promising HNADMs. Based on the literature (7), five commonly used carbon sources were selected as mentioned in the Materials and Methods section. Results showed that glucose (C_6_H_12_O_6_) favored the removal (84.6%) of ammonium nitrogen by strain AY4 more than other carbon sources, producing the highest cell growth (OD_600_, 2.1) (Fig. 3A). In contrast, when nitrate was used as a nitrogen source, AY4 preferred sodium succinate (C_4_H_4_Na_2_O_4_) as the carbon source (Fig. 3B).

#### Carbon-to-nitrogen ratio

In addition to carbon sources, C/N is equally important as it affects strain growth, electron supply, and energy pathway. As shown in Fig. 3C and D, the most suitable C/N was found to be 6 for growth and nitrogen conversion by strain AY4 during nitrification and denitrification processes, respectively. At optimal C/N conditions, 83.7% and 87.6% removal rates of ammonium and nitrate nitrogen were achieved, respectively, with higher cell densities.

#### Initial pH

pH level can affect the catalase activity of cells and cell membrane permeability. For the majority of HNADMs, the optimal pH range is 7 to 8 (7). A pH gradient study to find the optimal pH range for AY4 has been done, and results are presented in Fig. 3E and F. When ammonium nitrogen was used as the nitrogen source, ammonium nitrogen removal and OD_600_ reached 91.8% and 2.43, respectively, when the initial pH was adjusted to 8. In contrast, pH change during culture after inoculation with strain AY4 revealed that the pH of the medium started to decrease at 12 hours of incubation, reaching a minimum of 5.8, and gradually increased, reaching 8.8 at the end of incubation. In contrast to ammonium nitrogen, the optimum initial pH for the aerobic denitrification process of nitrate nitrogen was 7, and the nitrogen removal rate reached up to 92.3%.

### Temperature

Incubation temperature is a critical factor affecting the growth rate and enzyme catalytic activity of any bacteria. As shown in Fig. 3G and H, strain AY4 showed the highest ammonium nitrogen and nitrate nitrogen removal as 92.2% and 94.6%, respectively, at the optimum incubation temperature of 30°C. Strain AY4 was effective in removing nitrogen in the range of 20°C–35°C; and even at 10°C, it was still efficient in removing 56.7% and 67.8% of ammonium and nitrate nitrogen, respectively (Fig. 3G and H).

### Dissolved oxygen

The nitrification process of ammonium nitrogen requires involvement of oxygen, but the traditional denitrification process is an anaerobic process making DO, a key factor in nitrogen removal. The shaker/agitation speed changes the level of DO in culture medium. Hence, the influence of DO on the growth and nitrogen removal with AY4 was investigated by changing the shaker speed.

As shown in Fig. 3I and J, the preferred agitation speed for strain AY4 in both nitrification of NH_4_^+^-N and denitrification of NO_3_^−^-N was kept at 180 rpm (shaker speed). At 180 rpm (DO, 8.4 mg/L), removal of NH_4_^+^-N and NO_3_^−^-N increased to 98.5% and 99.8%, respectively, and the cell densities reached 2.5 and 3.5, respectively.

### Real wastewater treatment

**Fig 4:**
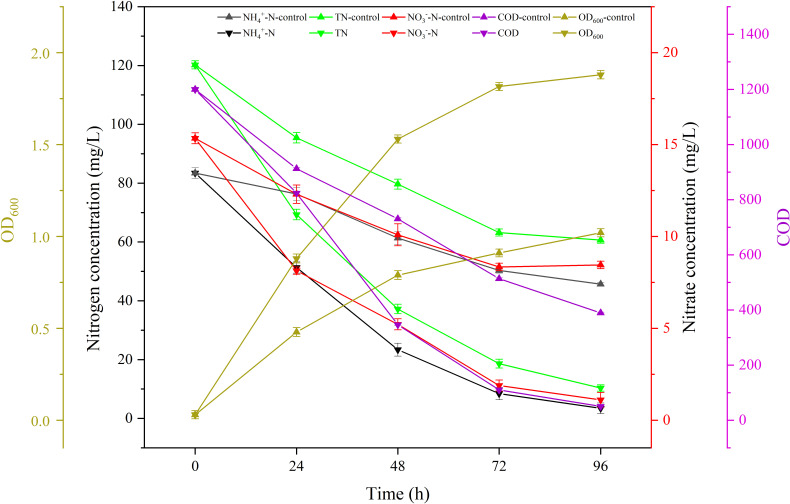
Characterization of nitrogen removal and bacterial growth during treatment of real wastewater with strain AY4. Data were analyzed using Duncan’s multiple comparison test. Different superscript characters in the bars indicate significant differences (*P* < 0.05)

Strain AY4 was introduced to real farm wastewater and characterized for the removal of NH_4_^+^-N, NO_3_^−^-N, and COD from the samples. They were obtained from a large-scale animal farm in Cangzhou, China, and the solids were removed by wet and dry sorting before the experiment. As shown in Fig. 4, compared with the control, the concentration of ammonium nitrogen in the experimental group decreased rapidly within 48 hours after AY4 inoculation, from 83.4 to 23.4 mg/L initially, and to 3.5 mg/L by 96 hours. Meanwhile, the ammonium nitrogen concentration in AY4 lacking a control group minimally decreased to 45.6 mg/L after 96 hours under the same culture conditions. TN in the experimental and control groups also showed different trends; TN in wastewater was reduced to 10.3 mg/L in the experimental group, which was 83.0% lower than that in the control group (60.7 mg/L). The level of COD in the experimental group decreased to 50.3 mg/L in 96 hours (removal rate 95.8%), while in the control group, the value was 389.2 mg/L. The removal trends of NH_4_^+^-N, NO3—N and TN were positively correlated with the density of bacteria in the wastewater. The bacterial density at OD_600_ for the experimental group reached up to 1.9, and the removal of NH_4_^+^-N, NO_3_^−^-N, and TN in the farm wastewater reached 95.8%, 91.4%, and 92.7%, respectively. In addition, a negative control group was set up after autoclaving the wastewater samples and remained uninoculated for 96 hours. The experimental results revealed that values of NH_4_^+^-N, NO_3_^−^-N, TN, and COD remained unchanged in this negative control group.

**Fig 5:**
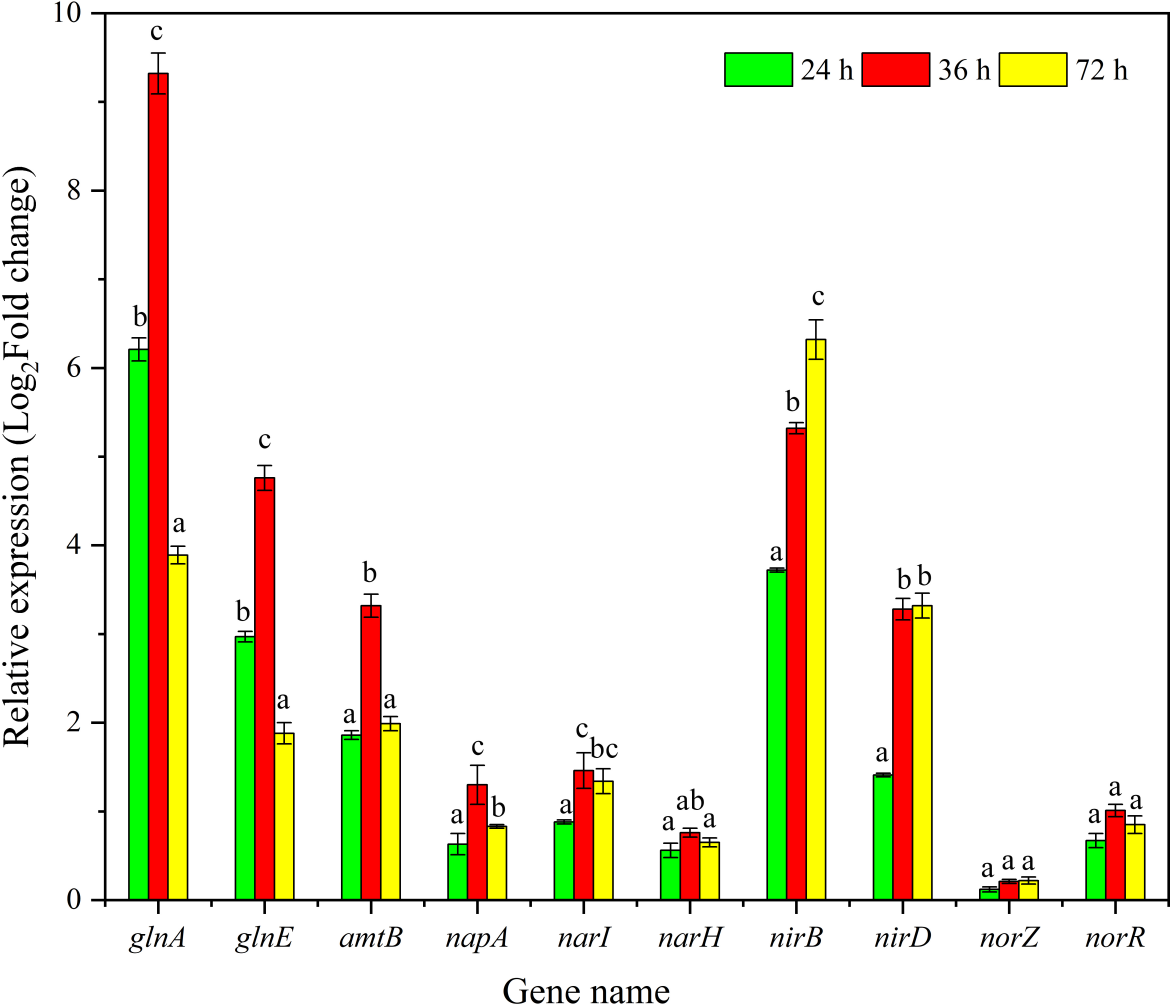
Relative expression of nitrogen removal genes of strain AY4 at 24, 36, and 72 hours in real wastewater treatment. Data represent means ± SE (*n* = 3). Means were analyzed using Duncan’s multiple comparison test. Different superscript characters in bar graphs indicate significant differences (*P* < 0.05)

To investigate the possible mechanism involved in nitrogen removal from wastewater by strain AY4, the transcript levels of *glnA*, *glnE*, *amtB*, *napA*, *narI*, *narH*, *nirB*, *nirD*, *norZ*, and *norR* genes were appraised and detected at 24, 36, and 72 hours of incubation, and the results are shown in Fig. 5. The *glnA* and *glnE* genes were for glutamine synthesis-related enzymes, and changes in their transcript levels were intimately related to ammonium nitrogen assimilation and conversion (29). The results demonstrated that the transcript levels of ammonium transport (*amtB*) and amino acid synthesis (*glnA* and *glnE*) were significantly upregulated after inoculation with strain AY4 for 24 hours incubation, and the highest level of *glnA* reached 7.3-fold (48 hours).

## DISCUSSION

The novel strain *P. stutzeri* AY4 has the same nitrogen removal capacity of ammonium and nitrate nitrogen as *Pseudomonas stutzeri* YG-24 (20) and *Pseudomonas stutzeri* YZN-001 (30) as found in previous literature. Some heterotrophic nitrifying-aerobic denitrifying bacteria (for example, *Acinetobacter calcoaceticus* HNR [31] and *Alcaligenes faecalis* NR [32]) catalyze the conversion of NH_4_^+^-N to NH_2_OH-N and then to gaseous-N during nitrogen removal, which is different from the metabolic process of NH_4_^+^-N conversion by nitrifying bacteria (7). Nitrite nitrogen and hydroxylamine were not detected in the removal of ammonium nitrogen of strain AY4 (Table 3; Fig. 2A). The reason for this may be that the concentration was too low or that they did not accumulate as intermediates and were rapidly converted to new substances (33), or it may be that the ammonium nitrogen was only assimilated to biological nitrogen without the nitrification reaction (34). Strain AY4 exhibited a similar ability as *Raoultella ornithinolytica* strain YX-4 to convert NO_3_^−^-N via the denitrification pathway under aerobic conditions (25). Nitrogen transformation characteristics suggested that strain AY4 could be a potential HNADM as previously reported *Alcaligenes faecalis* SDU20 (35) and *Pseudomonas stutzeri* (27).

Different from autotrophic microorganisms, HNADMs are able to assimilate and utilize organic carbon sources to accommodate growth and nitrogen removal. The experimental results revealed that glucose was more favorable to the utilization and removal of ammonium nitrogen by strain AY4 than other carbon sources (Fig. 3A), in contrast to sodium succinate, which was more favorable to enhance the denitrification process of nitrate nitrogen (Fig. 3B). Similar results were observed with the heterotrophic nitrifying bacterium *Pseudomonas aeruginosa* P-1 (36), where the most suitable carbon source was also glucose, as we have seen in the case of AY4. The choice of different carbon sources for HNADMs had also been reported for *R. ornithinolytica* strain YX-4 (25) because it was postulated that the carbon source not only provided energy for the growth of that bacterium but also provided electrons for the conversion of nitrogen (37).

Strain AY4 had efficient ammonium and nitrate nitrogen removal at low C/N (6.0), whereas HNADMs reported in the literature have different requirements for optimum C/N (7). Unlike strain *Alcaligenes faecalis* SDU20, which has an optimal C/N requirement of 10 (35), strain AY4 is similar to *Marinobacter* NNA5, which showed better growth and nitrogen conversion when C/N ranges from 6 to 8 (38). Substantial use of high-protein content feeds in the animal farming process causes low C/N in agricultural wastewater. As a result, in order to achieve efficient removal of excess nitrogen from such wastewater, enormous quantities of carbon sources are needed to accommodate the high volume of HNADMs performing denitrification. Assessment of HNADMs adapted to low C/N is more advantageous to keep the wastewater treatment cost in check and sustainable. Strain AY4 is capable of removing nitrogen at a low C/N condition, acknowledging a more economical approach toward bioremediation of wastewater.

HNADMs prefer a near-neutral (pH ~7.0) to a strong acidic or alkaline environment for growth, which could cause the bacterial surface to accumulate large amounts of charge and destroy the integrity of the cell membrane (39). The optimum initial pH for strain AY4 was in the range of 7–8. Similar results were found in the case of *Photobacterium* sp. NNA4 (40) and *P. stutzeri* SDU10 (23), where the optimal pH range was found to be 7–8, similar to AY4.

Temperature affects the efficiency of nitrification and denitrification by influencing the activities of intracellular and extracellular bioenzymes of HNADMs. The efficiency of nitrification was reduced by a doubling for every 10°C decrease in incubation temperature, and the efficiency of denitrification was similarly reduced by a doubling for every 4°C decrease in temperature (41). Although the optimal incubation temperature for strain AY4 is 30°C, it has the ability to remove both ammonium nitrogen and nitrate nitrogen in the range of 10°C–35°C, showing excellent temperature adaptability. Similar results were found in the case of *Pseudomonas balearica* RAD-17 (42) and *Serratia marcescens* W5 (21), where broad temperature adaptation was seen in both the strains for treating multiple real wastewaters. Additionally, their low-temperature adaptability shall be particularly beneficial in cold-region wastewater treatment.

Dissolved oxygen in the medium should not only meet the needs of bacterial growth but also serve as an electron donor in the denitrification process of nitrate nitrogen for electron transfer in the aerobic respiratory chain (43). Nitrite reductase (NIR) is sensitive to oxygen, and high DO inhibits the activity of NIR, and then it becomes a rate-limiting enzyme in the denitrification process of NO_3_^−^-N. The effect of oxygen on the nitrogen removal efficiency of strain AY4 was evaluated by adjusting the DO level in the medium through the shaker speed, and it was found that the most suitable shaker rate for both ammonium and nitrate nitrogen removal was 180 rpm. Similar to strain AY4, *Acinetobacter* sp. T1 (44) and *Photobacterium* sp. NNA4 (40) had an optimal shaker speed of 160 rpm and also exhibited high levels of dissolved oxygen tolerance. Differences in ammonium nitrogen and nitrate nitrogen also affect the differences in DO level requirements of HNADMs. *R. ornithinolytica* strain YX-4 (25) showed optimum ammonium nitrogen conversion at 180 rpm, but denitrification required 150 rpm, whereas strain AY4 was capable of removing both NH_4_^+^-N and NO_3_^−^-N efficiently at 180 rpm.

HNADMs have been successfully applied in wastewater treatment. *P. stutzeri* SDU10 was able to remove 97.6% and 94.2% of ammonium nitrogen and COD from the sewage in the application of pig farm wastewater treatment (23). Other applications of HNADMs include *Acinetobacter* sp. T1 (44), *Paracoccus denitrificans* Z195 (45), and *Serratia marcescens* CL1502 (22), all of which have been applied successfully in the treatment of nitrogenous wastewater. Strain AY4 also has the potential to be applied in the treatment of farm wastewater, and the experimental data showed that it was able to effectively remove NH_4_^+^-N, NO_3_^−^-N, and TN from farm wastewater, with removal rates of 95.8%, 91.4%, and 92.7%, respectively. The removal rates of ammonium nitrogen and nitrate nitrogen in the real farm wastewater inoculated with strain AY4 were up to 1.3 and 0.3 mg/L/h, respectively, which were much higher than the nitrogen removal rates of 0.6 and 0.1 mg/L/h in *R. ornithinolytica* strain YX-4 (25).

The level of transcription of ammonium nitrogen assimilation-related enzyme genes increased with increasing AY4 cell density within the range of 0–72 hours, suggesting that ammonium nitrogen is preferentially synthesized into proteins used for bacterial growth. The upregulation of the transcript level of the *amtB* gene, which is responsible for the ammonium ion transport channel in the cell membrane, facilitates the entry of more extracellular ammonium nitrogen into the cell, so that it can be involved in the metabolism of amino acids to synthesize more organic nitrogen, which is consistent with the upregulation of the transcript level of *glnA* and *glnE*. The high concentration of ammonium nitrogen can upregulate the genes related to nitrogen metabolism, which may be the reason for the efficient conversion of ammonium nitrogen by strain AY4. As with strain AY4, the transcript levels of *glnA* and *glnE* were also significantly upregulated in *R. ornithinolytica* strain YX-4 (25). The *nirB *and *nirD* (nitrite reductase) significantly increased in transcript levels, which contributed to the efficiency of nitrite conversion and reduced accumulation during metabolism. The transcript levels of *norZ* and *norR* (nitric oxide reductase) did not significantly increase, and it was hypothesized that most of the nitrate nitrogen might be converted into nitric oxide and escaped the medium rather than forming nitrogen gas for stripping. This result was different from that of *Acinetobacter oleivorans* AHP123 reported previously, in which the transcript level of the nitric oxide reductase gene (*nor*) was significantly upregulated during denitrification of nitrate nitrogen during bioremediation (24). Changes in the transcript levels of key enzyme genes during the real wastewater treatment process can better elucidate the mechanism of nitrogen-containing wastewater removal of HNADMs.

### Conclusions

*P. stutzeri* strain AY4 capable of eliminating excess nitrogen from wastewater made it a potential HNADM showing 95.8%, 91.4%, and 92.7% of ammonium nitrogen, nitrate nitrogen, and TN removal from animal farm wastewater. Significant upregulation in *glnA*, *glnE*, *amtB*, *napA*, *narI*, *nirB*, and *nirD* transcript levels (associated with nitrogen assimilation and denitrification) was detected by qRT-PCR. Microbial bioremediation has become a priority to attain sustainable development goals (SDGs), as adopted by the United Nations. Findings of the present study certainly help in benefiting multiple levels of humankind, including clean water and sanitation (SDG6), climate action (SDG13), and life below water (SDG14).
